# Hospitalization Rates and Direct Medical Costs for Fever in a Pediatric Cohort in South India

**DOI:** 10.1093/infdis/jiab329

**Published:** 2021-11-23

**Authors:** Manikandan Srinivasan, Kulandaipalayam Natarajan Sindhu, Atrayee Nag, Arun S Karthikeyan, Ranjith Kumar Ramasamy, Malathi Murugesan, Dilesh Kumar, Santhosh Kumar Ganesan, Winsley Rose, Gagandeep Kang, Jacob John

**Affiliations:** 1 The Wellcome Trust Research Laboratory, Division of Gastrointestinal Sciences, Christian Medical College, Vellore, India; 2 Department of Child Health, Christian Medical College, Vellore, India; 3 Department of Community Health, Christian Medical College, Vellore, India

**Keywords:** hospitalization, incidence, health care, children, medical costs, antibiotics, India

## Abstract

**Background:**

Primary data on causes and costs of hospitalization are necessary for costing and cost-effectiveness analysis. Data on incidence and causes of hospitalization and consequent expenses among Indian children are limited.

**Methods:**

A cohort of 6000 children aged 0.5–15 years residing in urban Vellore was followed for 3 years, under the Vellore Typhoid Study, 2016–2017, and later under the Surveillance for Enteric Fever project, 2017–2019. Data on hospitalization events and associated antibiotic use, and direct medical costs for fever-related hospitalization of study children were obtained from caregivers through weekly follow-up by study field workers.

**Results:**

The incidence of hospitalization was 33 per 1000 child-years of observation. Children aged 0.5–5 years had the highest incidence of hospitalization. The top 5 infectious causes for hospitalization were acute undifferentiated fevers, respiratory tract infections, acute gastroenteritis, enteric fever, and dengue. The overall median cost of hospitalization for fever was 4243 (interquartile range, 2502–7215) Indian rupees (INR). An episode of dengue had a median cost of 5627 INR, followed by acute undifferentiated fevers and enteric fever with median costs of 3860 and 3507 INR, respectively.

**Conclusions:**

Hospitalization for fever is common in young children and impacts household finances in low-income Indian households.

In 2015, India accounted for about a fifth of the global under-5 mortality, with pneumonia and diarrhea as the leading causes [1]. In Indian children aged between 5 and 14 years, 58% of mortality was attributed to infectious diseases [2]. With children younger than 15 years constituting one-third of India’s population, there is high demand for health resources [3]. Reducing morbidity and mortality through investment in cost-effective interventions requires real-time data on the causes for hospitalizations, utilization pattern of the existing health care systems, in particular, inpatient services, and the expenses incurred by the household.

In 2018, the prevalence of all-cause hospitalization across age groups was estimated to be 29 per 1000 population in India (Tamil Nadu = 29–33 per 1000 population) [4]. Furthermore, 20% of hospitalizations reported at the district level were among children younger than 15 years [5]. Acute febrile illnesses, diarrhea and respiratory infections accounted for nearly three-fourth of all-cause hospitalization in these children [6]. In the National Sample Survey (NSS), male children, those from urban settings, and higher socioeconomic strata were more likely to be hospitalized [4].

According to the National Health Accounts 2016–2017, out-of-pocket expenditure (OOPE) by Indian households has fallen from 69.4% in 2004–2005 to 58.7%, following the introduction of government health insurance schemes [7]. The median OOPE towards hospitalization in children younger than 15 years ranged between 12 931 and 16 352 Indian rupees (INR), with the ratio of OOPE to total annual consumption expenditure (household spending on goods and services) ranging between 0.15 and 0.20 [5]. The private sector, which accounts for 61% of hospitalizations, for all age groups in urban India, had a mean OOPE of 38 822 INR, which is 8 times higher than the OOPE incurred in government facilities with a mean OOPE of 4837 INR [4]. This is a major concern for poorer families that end up in catastrophic health expenditure. In the National Health Policy 2017, the Indian Government has set a target of a 25% reduction in household catastrophic health expenditure, defined as health expenditure > 10% of total monthly food consumption or >40% of monthly non-food consumption expenditure, by 2025 [8]. To achieve this goal, documenting costs incurred by households for different forms of illnesses and care is essential. In children, most hospitalizations are due to infectious diseases and in addition to medical management expenses, a focus on understanding the use of antibiotics is important. At private hospitals, 77% of common pediatric illnesses were managed with antibiotics, particularly for suspected enteric fever (92%), respiratory tract infections (87%), fever of unknown origin (80%), and acute gastroenteritis (68%) [9]. Medication costs, predominantly driven by antibiotics, accounted for 24% of OOPE during hospitalization in India [4]. With OOPE correlating with antimicrobial resistance in low- and middle-income countries, tracking expenditure on antibiotics could potentially provide a tool for measuring the quality of care over time [10].

Although the NSS collects data on hospitalization costs, the survey is subject to recall bias because data are collected retrospectively for the previous year. In this study, we prospectively collected the incidence and causes of hospitalization, antibiotic usage, and associated direct medical costs for fever-related hospitalizations in a cohort of 6000 children at Vellore aged between 6 months and 15 years followed-up for febrile illnesses.

## METHODS

### Study Setting, Participants, and Data Collection

The Vellore Typhoid Surveillance (VTS) study in India cohort was established in October 2016 by investigators at the Christian Medical College (CMC), Vellore, to study the incidence of enteric fever in children in urban Vellore. In October 2017, this cohort was included within the Surveillance for Enteric Fever in India (SEFI) project, with active surveillance for fever in children established at 4 sites in India: Vellore, Pune, Kolkata, and Delhi. The overarching aim of SEFI was to estimate the incidence of enteric fever in Indian children. The SEFI protocol has been described in detail elsewhere [11]. Overall, a cohort of approximately 6000 children aged between 6 months and 15 years were actively followed up at the Vellore site every week between 2016 and 2019 to identify fevers. Blood culture was performed for any child with a fever of 3 or more days.

To estimate the incidence of all-cause hospitalization, we used data from 6070 children in the VTS and SEFI studies between 2016 and 2019. These children were enrolled from 4 contiguous semiurban settlements of Vellore: Chinnallapuram, Kaspa, Ramnaickanpalayam, and Vasanthapuram, with population characteristics described elsewhere [11]. The community consists of families that predominantly belong to the lower socioeconomic strata. Outpatient services for children are provided free by a study clinic. Children also seek care from the general private practitioners located in and around the area. Children requiring hospitalization may be admitted to several nursing homes or 4 large hospitals, 2 governmental and 2 nonprofit private hospitals. The 2 private organizations, CMC and Sri Narayani Hospital, operate on a no-profit, no-loss basis but also provide concessional care for poor patients. The 2 government health facilities offer state health care services free, with no charges for admission, consultation, diagnostics, drugs, or procedures. CMC also caters to the health care needs of this community through its 2 secondary-care outreach hospitals, the Community Health and Development Hospital (CHAD) and the Low-Cost Effective Care Unit (LCECU), both located within approximately 5 km radius of the surveillance area.

For the surveillance, the primary caregivers of the study children were contacted weekly to capture information on fever, other common morbidities, and hospitalization in the preceding week. In case of hospitalization of a study child, copies of discharge summaries and hospital expenditure bills were requested. Details extracted from the summaries and bills included duration of hospitalization, final diagnosis at discharge, antibiotic usage with reference to type and duration, and the overall costs incurred. For hospitalizations at CMC, whenever discharge summaries were not available with the patient, they were retrieved from electronic hospital records. For hospitalizations in facilities other than CMC where a discharge summary was not available, details of hospitalization were obtained from the primary caregivers. Throughout the surveillance period, to ensure no hospitalizations were missed during surveillance, trained study nurses visited the main hospitals daily to track all hospitalizations. Fever-related hospitalizations were broadly classified as acute undifferentiated fevers, respiratory tract infections, acute gastroenteritis, enteric, and dengue fever, based on the final diagnosis in the discharge summary. Acute undifferentiated fevers were defined as those fevers where there was no evidence of a systemic focus/foci of infection, being documented by the examining physician as acute febrile illness or fever without a focus/foci of infection, or fever for evaluation (following which an absent focus/foci of infection was confirmed).

From CMC, costs related to investigations, drugs, and consumables; inpatient charges that included bed charges; and consultation/professional fee for treatment and procedures could be obtained from the hospital bills. For hospitalization at private hospitals other than CMC, only the overall cost was captured, as costs were not categorized.

### Statistical Analysis

The period of follow-up for hospitalization between 2016 and 2019 was divided as follows: year 1, October 2016–October 2017; year 2, November 2017–October 2018; and year 3, November 2018–October 2019. The incidence rate of hospitalization was calculated by the number of hospitalization events over child-years of observation (CYO). CYO were deduced by presetting the data to a survival-time set data mode (stset) on STATA 14.0 (StataCorp 2015, Stata Statistical Software release 14). The period for which surveillance was not available, most commonly for travel out of the study area, and duration of hospitalization was subtracted from CYO for the child. Person-time contribution under age-specific intervals in the cohort was estimated by dividing the study follow-up period into 0.5 to <5 years, 5 to <10 years, and 10 to <15 years, where contribution by an individual participant within these intervals was measured based on the age of the child at the time of entry into the age interval over the course of the study. In the analysis, multiple events were allowed for each child, with the provision for children moving higher up in the age intervals over time. The incidence rates of hospitalization were expressed per 1000 CYO. Categorical variables that included cause and place of hospitalization and antibiotics prescribed were expressed as percentages. Graphs were used to represent the categories of final diagnoses for hospitalizations and time trends. Direct medical costs for fever-related hospitalizations were stratified by place of hospitalization and diagnoses categories and were summarized using median and interquartile range (IQR).

VTS and SEFI were approved by the institutional review board of Christian Medical College. Written informed consent from the primary caregivers of all children, and an assent from children aged >12 years were obtained.

## RESULTS

**Figure 1.**
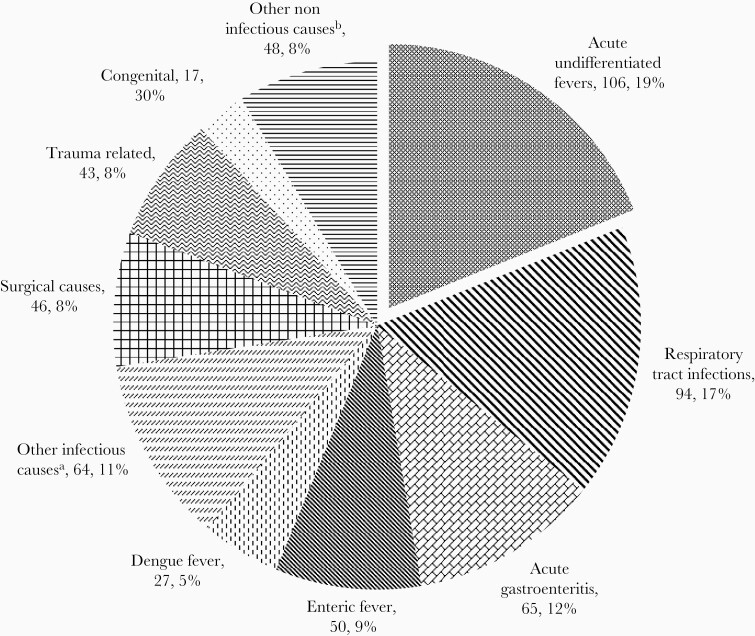
Causes of hospitalization in the Vellore cohort between 2016 and 2019 (n = 560). ^a^Includes infections of genitourinary system, viral hepatitis, mumps, meningitis, skin infections, tuberculosis, and other infectious conditions. ^b^Includes seizure disorders, poisoning, renal diseases, and others.

**Figure 2.**
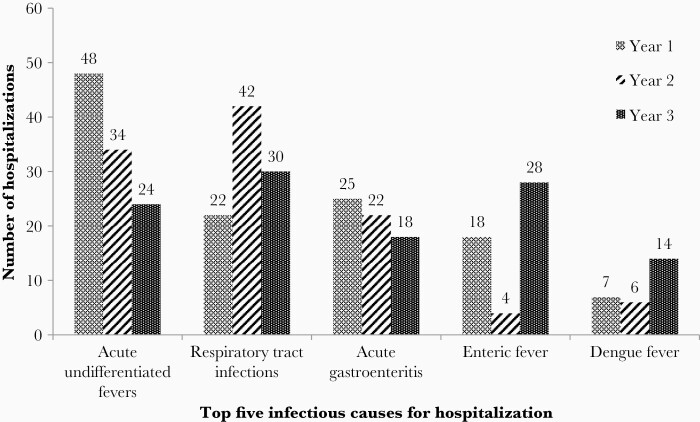
Year-wise distribution of top 5 infectious causes of hospitalization between 2016 and 2018 (n = 342). Year 1 = October 2016–October 2017; year 2 = November 2017–October 2018; year 3 = November 2018–October 2019.

There were 560 hospitalizations among the 6070 children between 2016 and 2019, with an incidence of hospitalization of 32.9 (95% confidence interval, 30.2–35.7) per 1000 CYO (Table 1). The incidence of hospitalization was higher among male children (35.4 and 30.2 per 1000 CYO in male and female children, respectively, *P* = .032). Age-stratified incidence showed that hospitalization was highest in children younger than 5 years as compared to those in the 10–15 years age group (51.2 and 25.6 per 1000 CYO, respectively, *P* < .0001). The main causes for hospitalization were acute undifferentiated fevers, respiratory tract infections, acute gastroenteritis, enteric fever, and dengue, and this pattern was observed in all 3 years (Figure 1 and Figure 2). The incidence of hospitalization for acute undifferentiated fevers, respiratory tract infections, acute gastroenteritis, enteric fever, and dengue were 6.2, 5.5, 3.8, 2.9, and 1.6 per 1000 CYO, respectively (Table 1). Respiratory tract infections (30%), acute undifferentiated fevers (18%), and acute gastroenteritis (13%) were the leading causes for hospitalizations in children younger than 5 years. In 5–15 year olds, acute undifferentiated fevers (20%), enteric fever (11%), and acute gastroenteritis (11%) were the top 3 illnesses resulting in hospitalization.

**Table 1.** Incidence of All-Cause Hospitalization Over a 3-year Period in a Cohort of Children 0.5 to 15 Years of Age Recruited Into a Febrile Surveillance Study in Vellore, Tamil Nadu (n = 6760)

Variable	n	CYO	Incidence Rate per 1000 CYO (95% CI)
Overall all-cause hospitalization	560	17 044	32.9 (30.2–35.7)
All-cause hospitalization by year^a^			
Year 1	170	5357	31.7 (27.2–36.9)
Year 2	188	6014	31.3 (27.1–36.1)
Year 3	202	5673	35.6 (31.0–40.9)
Age-specific incidence of hospitalization			
<5 y	216	4276	50.5 (44.0–57.7)
5–9 y	195	6867	28.4 (24.6–32.7)
10–15 y	149	5901	25.3 (21.4–29.7)
Sex-specific incidence of hospitalization			
Male	307	8677	35.4 (31.6–39.6)
Female	253	8367	30.2 (26.7–34.2)
Socioeconomic status^b^			
Low	396	11 882	33.3 (30.2–36.8)
Middle	140	4538	30.8 (26.1–36.4)
High	17	508	33.5 (20.8–53.8)
Hospitalization for common morbidities, top 5 infectious causes (n = 342)			
Acute undifferentiated fevers	106	17 044	6.2 (5.1–7.5)
Respiratory tract infections	94	17 044	5.5 (4.5–6.7)
Acute gastroenteritis	65	17 044	3.8 (2.9–4.9)
Enteric fever	50	17 044	2.9 (2.2–3.9)
Dengue fever	27	17 044	1.6 (1.0–2.3)

Abbreviations: CI, confidence interval; CYO, child-years of observation.

^a^Year 1 = October 2016–October 2017; year 2 = November 2017–October 2018; year 3 = November 2018–October 2019.

^b^Data on socioeconomic status missing for 7 data points.

The incidence rates of hospitalizations across the 3 years of the study for enteric fever were 3.4, 0.7, and 4.9 per 1000 CYO, and for dengue fever the rates were 1.3, 1.0, and 2.5 per 1000 CYO. Of the 560 hospitalizations, 260 (46.4%) were at the not-for-profit private facilities while 257 (45.9%) hospitalizations were at government hospitals (Table 2). The median duration of hospitalization was 4 days (IQR, 3–6 days).

**Table 2.** Hospital Facility Utilized and Duration of Hospitalization in the Cohort (n = 560)

Health Care Facility	Value
Secondary-care facility^a^ (n = 360)	
Government secondary-care hospital	180 (32)
CHAD^b^	91 (16.3)
LCECU^b^	63 (11.3)
Private hospital	26 (4.6)
Tertiary-care facility^c^ (n = 189)	
CMC^b^	106 (18.9)
Government tertiary-care and referral hospital	77 (13.8)
Private hospital	6 (1.1)
Hospitalization in more than a single facility for an episode of illness	11 (2)
Duration of hospitalization, d, median (IQR)	4 (3–6)

Data are No. (%) except where indicated.

Abbreviations: CHAD, Community Health and Development; CMC, Christian Medical College; IQR, interquartile range; LCECU, Low-Cost Effective Care Unit.

^a^Secondary-care facility: A hospital serving a population of 100 000 in a defined geographical area and serves as the first referral center from a primary health care facility.

^b^Not-for-profit private organization.

^c^Tertiary-care facility: Medical college/multispeciality hospitals that are the highest referral centers from primary and secondary health facilities.

**Figure 3.**
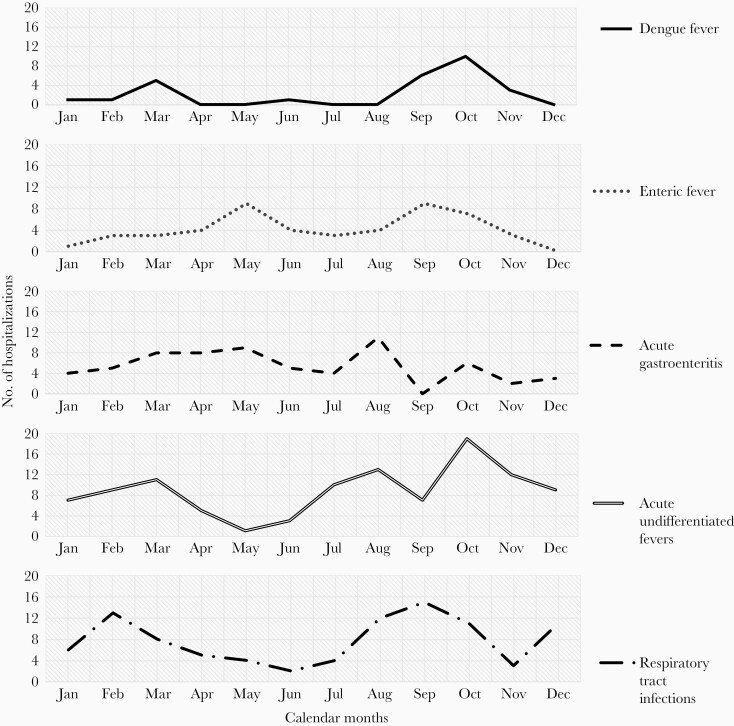
Cumulative distribution of top 5 infectious causes for hospitalization, between 2016 and 2019 represented by month, showing seasonal patterns (n = 342).

A seasonal variation in causes for hospitalizations was observed. Acute gastroenteritis sharply peaked in August, while respiratory tract infection-related admissions showed a bimodal peak in February and September (Figure 3). Both acute undifferentiated fevers and dengue fever-related admissions were highest in October. Admissions for enteric fever showed a bimodal peak in May and September.

Of the 560 hospitalizations, data on direct medical costs were available for 158/429 fever-related hospitalizations (three-fourth of the fever-related hospitalizations that did not have data on costs were at government health facilities where health care is provided free of cost, with costs being incurred only towards consumables/investigations not available in-house. For a proportion of hospitalizations at study facilities and private health care facilities, the study team could not retrieve medical bills from the families even after multiple attempts). The median cost of a fever-related hospitalization, irrespective of the place of admission, was 4243 INR (IQR, 2502–7215) (Table 3). The median cost of hospitalization at private health facilities and nonprofit tertiary-care setting was 9000 INR (IQR, 5000–22 000) and 9630 INR (IQR, 5070–13 879), based on 7 and 45 hospitalization episodes, respectively, where information on hospital expenses was available. Dengue fever resulted in the highest overall median cost of 5627 INR, followed by acute undifferentiated fevers and enteric fever with a median cost of 3860 and 3507 INR, respectively. The median cost incurred towards investigations was the highest for dengue fever (2651 INR) followed by acute undifferentiated fevers (1509 INR). Dengue fever also had the highest median cost of 1613 INR towards overall inpatient charges. For costs incurred towards medicines during hospitalization, acute gastroenteritis (1564 INR) followed by enteric fever (1418 INR) had higher median costs.

**Table 3.** Direct Medical Costs^a^ of Fever-Related Hospitalizations Incurred at Different Health Facilities (n = 158/560^b^^,^^d^)

	n^c^	Investigation Costs	Inpatient Costs Inclusive of Consumables	Medication Costs	n^d^	Total Costs Incurred Towards Hospitalization
Total	151	1625 (570–2817)	1050 (240–1710)	1247 (791–2130)	158	4243 (2502–7215)
**Facility-wise direct medical costs**						
Facility						
CMC^e^	45	2426 (1270–4160)	2135 (1050–5565)	3031 (1334–4462)	45	9630 (5070–13 879)
CHAD^e^	69	1350 (740–2170)	1090 (910–1450)	972 (604–1414)	69	3507 (2586–4878)
LCECU^e^	31	667 (0–2027)	0	1013 (731–1428)	31	1830 (1043–3021)
Hospitalization in more than a single facility for an episode of illness	6	2223 (1895– 3830)	3875 (1440–12 540)	1680 (940–5231)	6	15 445 (4312–26 001)
Private hospitals	0	…	…	…	7	9000 (5000–22 000)
**Direct medical costs for the top 5 infectious causes for hospitalization in the Vellore cohort**						
Cause						
Acute undifferentiated fevers	12	1509 (912–2422)	570 (0–1223)	1108 (736–1485)	15	3860 (3052–6542)
Respiratory tract infections	36	1039 (284–1613)	873 (0–1753)	995 (712–2013)	36	2456 (1527–6723)
Acute gastroenteritis	5	107 (0–1720)	0 (0–490)	1564 (928–1687)	6	3475 (1687–7923)
Enteric fever	36	1068 (60–2105)	1160 (415–1450)	1418 (990–2253)	37	3507 (2825–5335)
Dengue fever	18	2651 (1895–3330)	1613 (1050–2855)	1287 (761–2185)	19	5627 (4357–11 536)

Data are median (IQR).

Abbreviations: CHAD, Community Health and Development; CMC, Christian Medical College; IQR, interquartile range; LCECU, Low-Cost Effective Care Unit.

^a^All costs calculated in INR (Indian rupee rate).

^b^Of the 560 overall hospitalizations, data on direct medical costs for hospitalizations was available for 158 children; 371 hospitalizations did not have data on direct medical costs of which 258 hospitalizations were at government health facilities where treatment is free of cost and 113 admissions were at health facilities of CMC and other private hospitals for which medical bills were not available. Additionally, 31 hospitalizations for which data on direct medical costs were available were not considered for this analysis as these hospitalizations were surgery-related (17), trauma (5), congenital (3), malignancy (2), chronic diseases (2), tonsillitis associated without fever (1), and seizure disorder (1).

^c^Includes those records with individual medical bills available for calculation of direct medical costs.

^d^Derived from medical bills available as well as history from primary caregiver for bills that were not available.

^e^Not-for-profit organization.

A total of 367 (65.5%) of the 560 inpatient admissions received antibiotics, with 713 antibiotic prescriptions being documented overall for the 367 admissions (Table 4). The most common class of antibiotics used was beta-lactam antibiotics (475, 66.6%), with third-generation cephalosporin contributing to two-third of these prescriptions. Furthermore, third-generation cephalosporins accounted for 321/713 (45%) of the overall antibiotic prescriptions.

**Table 4.** Antibiotic Prescription During Hospitalization (n = 560)

Prescription	n^a^ (%)	Median No. of Antibiotics Used (IQR)
No. of antibiotics		
Hospitalizations with antibiotics prescribed	367/560 (65.5)	…
Children given 1 antibiotic	152/367 (41.4)	…
Children given 2 antibiotics	118/367 (32.2)	…
Children given 3 or more antibiotics	97/367 (26.4)	…
Total number of antibiotics prescribed for the 367 hospitalizations	713	…
Commonly used antibiotics (n = 713^b^)		
Beta-lactam group^c^	475 (66.6)	…
Amino glycosides	81 (11.4)	…
Macrolides	56 (7.9)	…
Tetracyclines	38 (5.3)	…
Fluoroquinolones	18 (2.5)	…
Others^d^	45 (6.3)	…
Top 5 infectious causes of hospitalizations^e^ (n = 342)		
Acute undifferentiated fevers	72/106 (67.9)	1 (0–2)
Respiratory tract infections	80/94 (85.1)	2 (1–3)
Acute gastroenteritis	31/65 (47.7)	0 (0–1)
Enteric fever^f^	49/50 (98)	2 (2–3)
Dengue fever	16/27 (59.3)	0 (0–1)

Abbreviation: IQR, interquartile range.

^a^Number of antibiotics prescribed among the number of hospitalization prescriptions retrieved during the surveillance.

^b^Events are not exclusive of each other; includes children who were on multiple antibiotics.

^c^Third-generation cephalosporins were 321/475 (67.6%) antibiotic prescriptions.

^d^Others included nitroimidazoles, glycopeptides, oxazolidinones, sulphonamides, first- and second-generation cephalosporins, polyenes, carbapenems, and nitrofuran.

^e^Diagnosis based on the final diagnosis mentioned in the discharge summary.

^f^Thirty-five were blood culture confirmed, with the remaining 15 cases being diagnosed as clinical enteric fever.

Antibiotic usage during hospitalization was highest among admissions attributed to enteric fever (49/50, 98%), respiratory tract infections (80/94, 85.1%), and acute undifferentiated fevers (16/27, 59%) (Table 4). The median costs incurred towards antibiotics during a fever-related hospitalization was 416 INR (IQR, 169–802). The ratios of median antibiotic costs to total direct medical and medicine-related costs for a fever-related hospitalization were 0.10 and 0.33, respectively. Analysis using available costing data points for antibiotics used during hospitalization showed that acute gastroenteritis (3/65) and enteric fever (35/50) had higher median costs of 576 and 569 INR, respectively. Although fewer children received antibiotics for acute gastroenteritis, third-generation cephalosporins were used for both acute gastroenteritis and typhoid. Acute undifferentiated fevers (9/106), respiratory tract infections (32/94), and dengue fever (10/27) admissions had relatively lower median costs incurred towards antibiotics of 392, 213, and 174 INR respectively (data not presented).

## Discussion

The incidence of hospitalization in children aged between 0.5 and 15 years was 33 per 1000 CYO in an urban setting in southern India. Children aged between 0.5 and 5 years were most likely to be hospitalized. The top 3 causes of hospitalization in young children were respiratory infections, acute undifferentiated fever, and acute gastroenteritis, while in older children, acute undifferentiated fever, enteric fever, and acute gastroenteritis predominated.

The recent round of NSS between 2017 and 2018 on 113 823 families reported a hospitalization incidence of 27 per 1000 population in children younger than 5 years, this is half the incidence obtained in our prospective cohort [4]. This difference can be explained by the NSS being based on a 365-day recall, while the incidence in our cohort was obtained from 3 years of weekly surveillance. Nearly, two-thirds of the cohort belonged to the lower socioeconomic strata, with approximately 80% of the study households depending on the public distribution systems for drinking water, where drinking water contamination has been documented [12, 13]. About 20% of these children live in households without access to improved sanitation (data not shown). Thus, given that a predominant portion of this urban cohort at Vellore resides in a setting with poor water, sanitation and hygiene, probably accounted for the high morbidity and hospitalizations [14].

A distinct seasonal pattern was observed for acute gastroenteritis, acute undifferentiated fevers, and dengue from August to October, which corresponds to the monsoon season. While the rise in cases of acute gastroenteritis could be due to contamination of drinking water supplies following rains, the surge in dengue hospital admissions post-monsoon could probably be due to the increased vector breeding in the community [15–17]. Notably, enteric fever admissions peaked between May and September, which is in concordance with available data favoring typhoid transmission during extremes of climatic conditions [18].

The incidence of hospitalization was higher in year 1 and 3 compared to year 2 of surveillance. This can be explained by the observed surge in cases of typhoid fever and a dengue outbreak [16] in the community in year 1 surveillance, and outbreaks of hepatitis A [13] and typhoid fever (submitted for publication) in year 3 [13, 16]. As a result of these outbreaks, severe cases that required hospitalization during year 1 and 3 were relatively higher compared to year 2. It is to be noted that in our surveillance, there were no reports of scrub typhus or malaria among the fever-related admissions given that this is an area with low transmission for these diseases.

The antibiotic prescription rate in the Vellore cohort was estimated to be 66% among the hospitalized, which was almost twice the global estimate [19]. Furthermore, studies across India, have shown that antibiotic prescription rates among pediatric inpatients were high and varied between 44% and 84% [9, 20, 21]. This higher antibiotic use could be because pediatric hospitalizations in India are predominantly of infectious origin and in a majority of the cases empirical antibiotic prescriptions are made while investigating the etiology, especially in resource-constrained settings due to lack of facilities and infrastructure [22, 23]. In this cohort, respiratory tract infections followed by acute undifferentiated fevers were the important drivers for antibiotic use in hospitalized children, corresponding to the findings from other Indian studies [9, 20, 21]. Thus, in the efforts to establishing antibiotic stewardship, it is important to implement and improve diagnostics that can differentiate infections of viral and bacterial etiologies, in cases of common pediatric morbidities such as respiratory infections and undifferentiated fevers, this going on to define the rationale for antibiotic usage in febrile children. With reference to the class of antibiotics, studies have shown that cephalosporins are the most common antibiotics used for hospitalized children in India, in line with the findings from our study [9, 20, 21]. High cephalosporin use, in the context of emerging evidence of ceftriaxone-resistant *Salmonella* Typhi strains in India, raises the need to focus on alternative strategies such as typhoid vaccination to bring down the infectious disease burden in children [24–26].

The direct medical costs for fever-related hospitalization were high, with costs per hospitalization in a nonprofit tertiary-care facility of 9630 INR. Comparing our findings to a non-profit hospital in the East Indian state of Odisha, we noted that direct medical costs for febrile hospitalization was approximately 7000 INR at Odisha, slightly lower than our estimate [27]. Furthermore, the Odisha study found that cost of diagnostics was a major driver of direct medical costs in febrile hospitalization, whereas we found that medicine-related costs was the highest contributor in a nonprofit tertiary-care setting [27]. However, this difference could be due to different causes of fever in cases admitted to these facilities and their corresponding treatment. Dengue fever had the highest direct medical costs of 5627 INR among all febrile hospitalizations in our study, ranging between 4357 and 11 536 INR. Findings from other studies have also shown that direct medical cost for dengue to be high and range between 9000 and 60 000 INR in private care facilities, this depending on the clinical severity of the illness at presentation [28–30]. The higher costs incurred in dengue admissions could be explained by the costs incurred towards repeat investigations such as the serial monitoring of platelets, until discharge, as per the dengue treatment protocol. In the case of acute differentiated fevers and acute gastroenteritis, it is important to note that direct medical costs reported in our study are conservative estimates with limited generalizability, given that there were limited data points on costs for these illnesses.

Fever-related illnesses, especially enteric and dengue fever, contribute to a significant proportion of hospitalization in children in an urban setting such as Vellore in South India. With Indian households having to meet almost four-fifth of the expenses towards hospitalization from household savings or income, the cost of febrile hospitalizations in our cohort is high for low-income families [4]. Pragmatic interventions such as vaccination, use of reliable as well as rapid diagnostic tests, along with the expansion of health insurance schemes for poorer families should be considered. Furthermore, additional data from other urban settings of India with different health care access would add to data of hospitalization patterns in children and costs of treatment.

## Conclusions

Children aged between 6 months and 5 years, who are at the highest risk for infectious diseases, contribute to a major portion of hospitalizations in India. Apart from acute febrile illnesses, respiratory tract infections and diarrhea, enteric fever, and dengue are important causes for hospitalization. Given the increasing direct medical expenses and constraints on the already stressed health care systems in India, policymakers need to consider pragmatic interventions to address this burden. Some of the potential interventions that could be implemented include vaccination, which has been shown to be cost-effective, while concurrently improving water, sanitation, and hygiene [31, 32].

## Notes

***Acknowledgments.*** We thank the parents and the children of the Vellore Typhoid Surveillance and Surveillance for Enteric Fever in India study. The authors thank the supervisors, the field research assistants, and Ms Jackline for their immense help in data collection.

***Author contributions.*** G. K. and J. J. conceived the study, drafted the original protocol, and provided critical revision of the final draft. M. S. and K. N. S. were involved in the conduct of the study, statistical analyses, and wrote and revised the manuscript. A. N. and M. M. assisted in data collection. A. K., R. K. R., D. K., and S. K. G. helped in the statistical analyses. W. R. provided clinical support. All authors approved the final version of the manuscript; agree to be accountable for all aspects of the work in ensuring that questions related to the accuracy or integrity of any part of the work are appropriately investigated and resolved; and confirm that all persons designated as authors qualify for authorship and all those who qualify for authorship are listed.

***Supplement sponsorship.*** This supplement is sponsored by the Christian Medical College Vellore Association.

***Financial support.*** This work was supported by the Bill and Melinda Gates Foundation (grant number OPP1159351 to G. K.).

***Potential conflicts of interest.*** All authors: No reported conflicts of interest. All authors have submitted the ICMJE Form for Disclosure of Potential Conflicts of Interest. Conflicts that the editors consider relevant to the content of the manuscript have been disclosed.
